# Bioinformatics analysis of epitope-based vaccine design against the novel SARS-CoV-2

**DOI:** 10.1186/s40249-020-00713-3

**Published:** 2020-07-10

**Authors:** Hong-Zhi Chen, Ling-Li Tang, Xin-Ling Yu, Jie Zhou, Yun-Feng Chang, Xiang Wu

**Affiliations:** 1 Department of Metabolism & Endocrinology, Metabolic Syndrome Research Center, Key Laboratory of Diabetes Immunology, Ministry of Education, National Clinical Research Center for Metabolic Disease, The Second Xiangya Hospital, Central South University, Changsha, 410011 Hunan China; 2 Department of Laboratory Medicine, The Second Xiangya Hospital, Central South University, Changsha, 410011 Hunan China; 3 Hunan Institute of Parasite Disease, WHO Collaborating Center For Research and Control on Schistosomiasis in Lake Region, Yueyang, 414000 Hunan China; 4 Department of Forensic Medicine Science, Xiangya School of Basic Medicine, Central South University, Changsha, 410013 Hunan China; 5 Department of Parasitology, Xiangya School of Basic Medicine, Central South University, Changsha, 410013 Hunan China

**Keywords:** SARS-CoV-2, Epitope, Vaccine, Bioinformatics

## Abstract

**Background:**

An outbreak of infection caused by SARS-CoV-2 recently has brought a great challenge to public health. Rapid identification of immune epitopes would be an efficient way to screen the candidates for vaccine development at the time of pandemic. This study aimed to predict the protective epitopes with bioinformatics methods and resources for vaccine development.

**Methods:**

The genome sequence and protein sequences of SARS-CoV-2 were retrieved from the National Center for Biotechnology Information (NCBI) database. ABCpred and BepiPred servers were utilized for sequential B-cell epitope analysis. Discontinuous B-cell epitopes were predicted via DiscoTope 2.0 program. IEDB server was utilized for HLA-1 and HLA-2 binding peptides computation. Surface accessibility, antigenicity, and other important features of forecasted epitopes were characterized for immunogen potential evaluation.

**Results:**

A total of 63 sequential B-cell epitopes on spike protein were predicted and 4 peptides (Spike_315–324_, Spike_333–338_, Spike_648–663_, Spike_1064–1079_) exhibited high antigenicity score and good surface accessibility. Ten residues within spike protein (Gly^496^, Glu^498^, Pro^499^, Thr^500^, Leu^1141^, Gln^1142^, Pro^1143^, Glu^1144^, Leu^1145^, Asp^1146^) are forecasted as components of discontinuous B-cell epitopes. The bioinformatics analysis of HLA binding peptides within nucleocapsid protein produced 81 and 64 peptides being able to bind MHC class I and MHC class II molecules respectively. The peptides (Nucleocapsid_66–75_, Nucleocapsid_104–112_) were predicted to bind a wide spectrum of both HLA-1 and HLA-2 molecules.

**Conclusions:**

B-cell epitopes on spike protein and T-cell epitopes within nucleocapsid protein were identified and recommended for developing a protective vaccine against SARS-CoV-2.

## Background

An outbreak of infection caused by a novel coronavirus has spread worldwide rapidly [1, 2]. The World Health Organization (WHO) named the disease COVID-19, short for “coronavirus disease 2019”. As of 27 May 2020, the WHO reported a total of 5 488 825 COVID-19 cases and 349 095 deaths globally [3], which brought a great challenge to public health worldwide. Therefore, it is imminent to prevent and control this infectious disease.

The pathogen causing the new type of pneumonia was named as severe acute respiratory syndrome coronavirus 2 (SARS-CoV-2, also called 2019-nCoV) by the Coronaviridae Study Group (CSG) of the International Committee on Taxonomy of Viruses [4]. Its genome sequence has been released and reported by Chinese scientists and submitted to the GenBank database on 12 January 2020 [5]. Like severe acute respiratory syndrome associated coronavirus (SARS-CoV) and Middle East respiratory syndrome coronavirus (MERS-CoV), the other two viruses that caused severe epidemic problems in recent years, SARS-CoV-2 also belongs to β-coronaviruses family. Bats are proved to be their natural host [6]. At present, insufficient knowledge of the latency and contagiosity of SARS-CoV-2 increased the uncertainty of virus persistency. Specific therapeutic agents targeting the virus are currently not available. Vaccination is still the most economic and effective approach to prevent virus infection. The selection and design of protective immunogens against pathogens is a major challenge in vaccine development, especially for the newly emerging pathogens [7, 8]. Traditional methods based on lab experiments could not meet the needs of the pressing situation in the event of an outbreak [9]. Bioinformatics is an interdisciplinary field specialized in organizing, storing, and processing large amounts of data generated from biological experiments. Accumulation of large-scale immunological data gave rise to the field known as immunoinformatics, which provides insights into the mechanisms of immune function. As the genome and protein sequence information of SARS-CoV-2 is available, characteristics of the virus, as well as the epitopes presented in the pathogen, could be predicted by in silico analysis, which will greatly speed up the vaccine development [10–12].

**Fig. 1:**
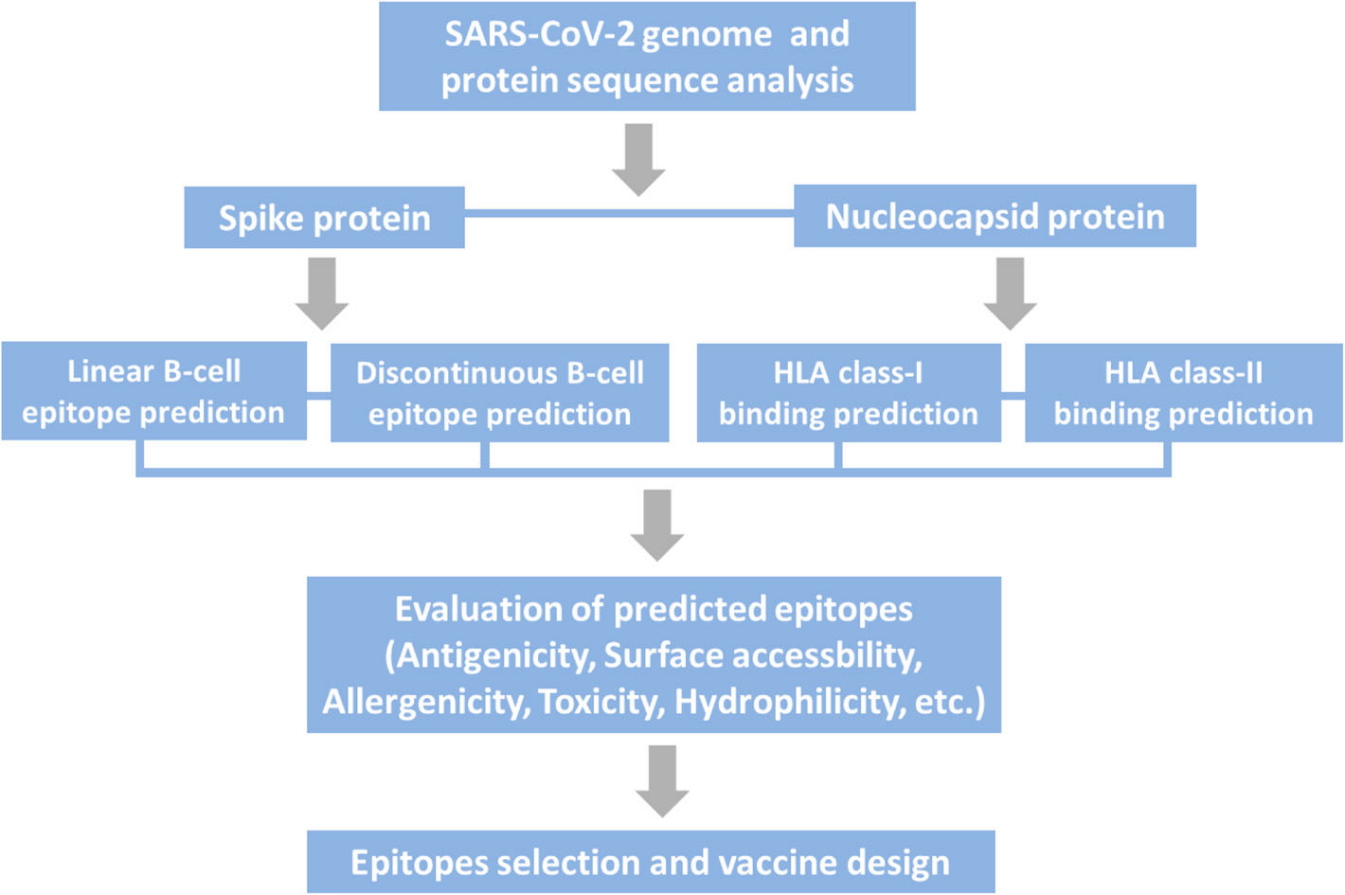
Study workflow. Suitable proteins of SARS-CoV-2 were selected at the first step for epitope prediction. The second step comprised of B- and T-cell epitope analysis with bioinformatics approaches. Epitope evaluation was followed and appropriate ones were chosen for vaccine design

The purpose of this study is to predict B-cell epitopes on spike protein and T-cell epitopes within nucleocapsid protein of SARS-CoV-2 by applying the bioinformatics methods and immunoinformatic tools. The step-by-step procedure of in silico analysis is depicted in Fig. 1. Epitopes information presented by this work may aid in developing a promising vaccine against SARS-CoV-2.

## Methods

### Data retrieval and sequence alignment

Protein sequences of SARS-CoV-2 were retrieved from the NCBI database (YP_009724390, YP_009724397). Clustal Omega is the current standard version of the Clustal family which was widely used for biological sequences alignment. The program uses seeded guide trees and Hidden Markov model engine to generate alignments [13]. In this study, alignment of protein sequences was performed on the EMBL-EBI server with Clustal Omega program. Conserved domains within predicted polypeptides of SARS-CoV-2 were analyzed with CD-search on the NCBI website.

### Linear B-cell epitope prediction

ABCpred [14] and BepiPred [15] servers were employed for B-cell epitope forecast. We used a threshold value of 0.85 to achieve a sensitivity between 95.5% and 99.5% for epitope prediction on ABCpred server [14]. The length of linear B-cell epitopes normally varies from 5 to 30 residues. In this study, we used the default window length of 16 to obtain the maximum accuracy of prediction [14]. Predicted epitopes were highlighted as sphere in SARS-CoV-2 spike protein structure viewed by the pymol molecular graphics system [16]. Surface accessibility of predicted peptides was evaluated with the recently resolved protein structure [17]. We utilized vaxijen2.0 server to analyze the antigenicity of chosen epitopes [18].

### Discontinuous B-cell epitope prediction

Prediction of discontinuous epitopes on spike protein (PDB ID: 6VSB chain B) was conducted via DiscoTope 2.0 server [19]. The parameter was set at − 1.0 which indicates 85% specificity and 30% sensitivity. This method is based on surface accessibility, residue statistics, and spatial information in a compiled data set of discontinuous epitopes discovered by X-ray crystallography of antigen/antibody complex structure. The contact number, propensity score, and disctope score for each amino acid are provided for conformation-based epitope prediction. Pymol was employed to illustrate the position of predicted epitopes on the 3D structure of SARS-CoV-2 spike protein recently resolved [17].

### T-cell epitope prediction

We used the free online service provided by IEDB to forecast T-cell epitopes within nucleocapsid protein binding to HLA-1 [20] or HLA-2 [21] molecule. A relatively small pool of HLA alleles covering the majority of the population, over 97 and 99% for class I and class II respectively, were chosen in the analysis [22, 23]. The sequences were given in plain format and the top 50% scoring peptides were retained for further analysis.

### Profiling and evaluation of predicted T-cell epitopes

Key features including digestion, mutation, toxicity, allergenicity, hydro and physiochemical properties were analyzed via vaxijen 2.0 [18], protein digest server [12], AllerTOP v2.0 server [24], and ToxinPred server [25]. Immunogenicity of predicted HLA-1 binding peptides was assessed by the Class-I Immunogenicity service provided on IEDB.

## Results

### Protein coding features of SARS-CoV-2 genome

**Fig. 2:**
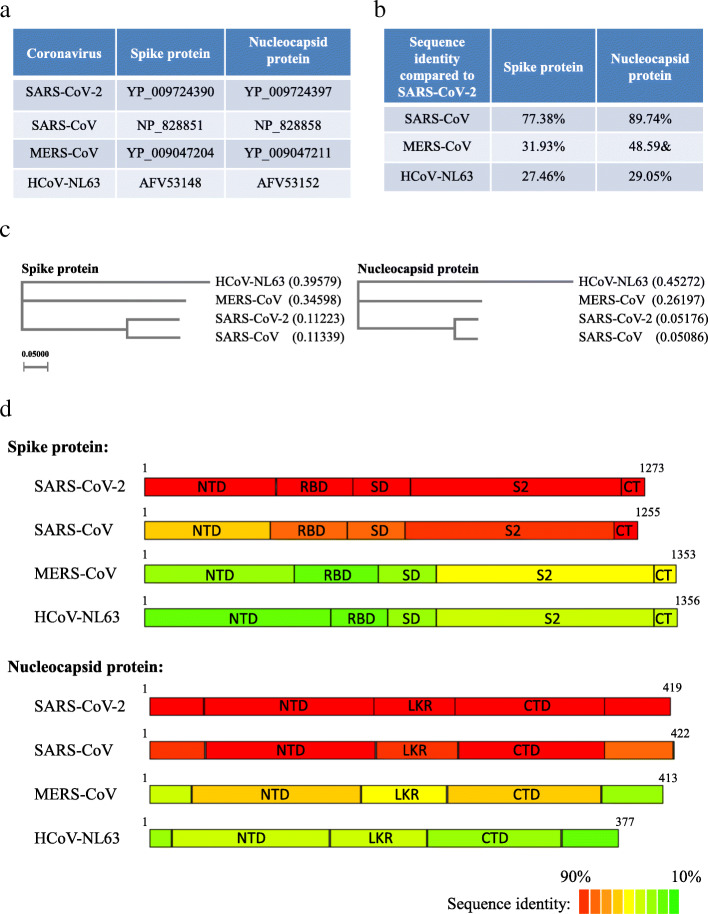
Spike and nucleocapsid protein in selected coronaviruses. **a** Accession IDs of spike protein and nucleocapsid protein in selected coronavirus. **b** Sequence identity of spike protein and nucleocapsid protein among selected coronavirus. **c** Phylogenetic analysis of target proteins of selected coronaviruses. **d** Sequence identity of subdomains of spike protein and nucleocapsid protein reflected by color (red color indicates high sequence identity and green color indicates low sequence identity)

A map of the predicted open reading frames (ORFs) is depicted in Supplementary Figure 1 based on the genome sequence of the virus Wuhan-Hu-1 isolate (NCBI reference sequence number: NC_045512.2). The genomic structure of SARS-CoV-2 shares characteristics that are also found in other coronaviruses including SARS-CoV, MERS-CoV, and HCoV-NL63. All these coronaviruses contain recognizable ORFs including the replicase (ORF1ab polyprotein), surface glycoprotein (spike protein), envelope protein, membrane glycoprotein, nucleocapsid protein, and several non-structure proteins (NSP). The conserved domains of proteins encoded by the SARS-CoV-2 genome are summarized in Supplementary Table 1. Spike protein mediates the specific binding of the virion to the receptor on the host cell membrane. The overall structure of spike protein is outside the virus particle [17]. Thus, it is an ideal target for B-cell epitope screening. Compared to spike protein, nucleocapsid protein is more conserved in selected coronaviruses (Fig. 2). Though unable to induce humoral immunity, nucleocapsid protein in SARS-CoV and MERS-CoV has been experimentally tested as a robust immunogen to induce cytotoxic T-lymphocyte (CTL)-mediated response [26, 27], which suggests nucleocapsid protein in SARS-CoV-2 could be a good candidate for T-cell epitope prediction.

### Sequence analysis of spike protein and nucleocapsid protein in selected coronaviruses

To better understand the characteristic of SARS-CoV-2, we compared its protein sequences with other selected coronaviruses. All protein sequences were downloaded from NCBI database with accession IDs shown in Fig. 2a. The total sequence identity and phylogenetic tree results were presented in Fig. 2b, c. Consistent with a recently published study [28], we found that both spike and nucleocapside proteins in SARS-CoV-2 are more closely related to that of SARS-CoV. The protein domains of spike and nucleocapsid proteins (Fig. 2d) were depicted based on previous studies on SARS-CoV [29, 30] and the protein alignment result in the current study (supplementary files S1 and S2). The amino acid sequence identity result confirmed a high similarity between SARS-CoV-2 and SARS-CoV. As anticipated, the nucleocapsid protein is more conserved among selected coronaviruses compared to spike protein.

### B-cell epitopes recognition

**Fig. 3:**
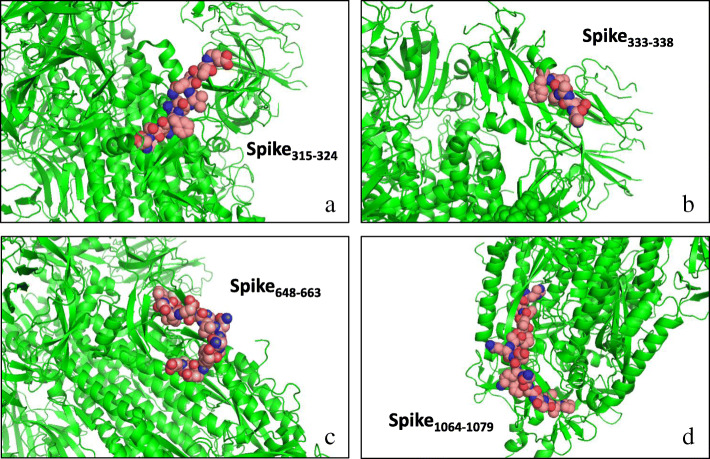
Site of B cell epitopes predicted in SARS-CoV-2 spike protein trimer. Spike_315–324_ (**a**), spike_333–338_ (**b**), spike_648–663_ (**c**), and spike_1064–1079_ (**d**) were highlighted in sphere in the protein structure. Colors of elements presented in the sphere of protein structure: carbon, tint; hydrogen, gray; nitrogen, blue; oxygen, red; sulfur, yellow

**Fig. 4:**
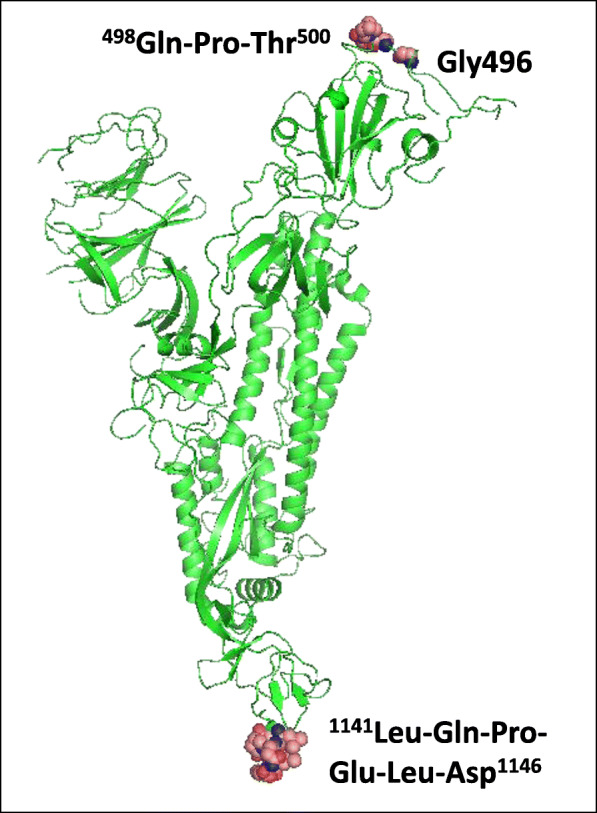
Site of B cells discontinuous epitopes predicted through DISCOTOPE 2.0 server on the structure of SARS-CoV-2 spike protein highlighted with cartoon representation

The full-length sequence of spike protein was scanned for putative sequential B-cell epitopes by two types of bioinformatics programs. A total of 28 non-overlapping peptides were identified by ABCpred server with the threshold set at 0.85 (Supplementary Table 2). For sequential B-cell epitopes prediction on BepiPred-2.0 server, a threshold value of 0.5 was applied and 35 peptides were predicted (Supplementary Table 3). Antigenicity was calculated by Vaxijen 2.0 server and peptides with the highest antigenicity scores were selected (Tables 1 and 2). The structure of SARS-CoV-2 spike protein was resolved recently with Cryo-electron microscopy (cryo-EM) [17], which could greatly facilitate the process of vaccine development. Predicted epitopes in Tables 1 and 2 were highlighted as sphere in monomer structure of spike protein viewed with pymol (Supplementary Figure 2). While most epitopes predicted were exposed on the surface of spike monomer, only epitopes Spike_315–324_ (TSNFRVQPTE), Spike_333–338_ (TNLCPF), Spike_648–663_ (GCLIGAEHVNNSYECD), Spike_1064–1079_ (HVTYVPAQEKNFTTAP) displayed good surface accessibility in spike trimer (Fig. 3 and Supplementary File: B-cell-epitope-animation.ppt), the pattern more likely exists in nature. Conformation-based B-cell epitopes were computed on DiscoTope 2.0 server [19]. A threshold value of − 1.0 was chosen for the computation, which corresponds to a specificity of 85% and a sensitivity of 30%. The contact number, propensity score, and disctope score for each amino acid that passed the threshold were presented in Table 3. The position of these residues was viewed with pymol and highlighted as sphere (Fig. 4). Processing with a combination of B-cell epitope scanning and peptide analysis forecasted 4 potent linear epitopes and 10 residues involved in discontinuous epitopes formation.

**Table 1:** B-cell epitope predicted via ABCpred server are presented along with their position and antigenicity scores

ABCpred	Position	Epitope sequence	Score	Antigenicity
Spike	406–421	EVRQIAPGQTGKIADY	0.85	1.3837
	648–663	GCLIGAEHVNNSYECD	0.90	0.8480
	898–913	FAMQMAYRFNGIGVTQ	0.88	1.3096
	1058–1073	HGVVFLHVTYVPAQEK	0.89	0.8847
	1064–1079	HVTYVPAQEKNFTTAP	0.88	0.8933
	1206–1221	YEQYIKWPWYIWLGFI	0.89	0.9510

**Table 2:** B-cell epitope predicted via BepiPred server are presented along with their position and antigenicity scores

BepiPred	Position	Epitope sequence	Antigenicity
Spike	315–324	TSNFRVQPTE	1.3571
	333–338	TNLCPF	1.2508
	372–397	ASFSTFKCYGVSPTKLNDLCFTNVYA	1.2880
	406–426	EVRQIAPGQTGKIADYNYKLP	1.3005
	1037–1045	SKRVDFCGK	1.7321
	1204–1209	GKYEQY	1.2821

**Table 3:** Discontinuous B-cell epitopes predicted through DiscoTope 2.0 server

Residue position	Residues name	Contact number	Propensity score	Discotope score
496	GLY	1	−0.693	−0.728
498	GLN	7	1.188	0.246
499	PRO	5	0.294	−0.315
500	THR	1	2.231	1.860
1141	LEU	5	−0.017	−0.59
1142	GLN	7	0.372	−0.476
1143	PRO	6	0.629	−0.134
1144	GLU	4	0.704	0.163
1145	LEU	5	0.171	−0.424
1146	ASP	4	0.724	0.181

### T-cell epitopes recognition

In our study, the IEDB server was utilized following prediction methods recommended (a combination of ANN, SMM, CombLib, and NetMHCpan EL methods for HLA-1 binding prediction, and a combination of NN-align, SMM-align, CombLib, Sturniolo, and NetMHCIIpan methods for HLA-2 binding prediction).

For HLA-1 binding peptide prediction, the top 50% scoring peptides were retained for further analysis. A total of 81 nonrepetitive peptides with ANN_IC50 value not higher than 500, indicative of stronger than medium binding affinity, were identified (Supplementary Table 4). Six peptides with the highest antigenicity scores by vaxijen 2.0 were chosen for next step processing. In this step, we screened all HLA-1 molecules being able to bind these peptides (Table 4). A similar strategy was applied for HLA-2 binding peptides prediction on the IEDB server and 64 peptides were identified as HLA-2 binding sequences (Supplementary Table 5). Six peptides with the highest antigenicity scores were selected for HLA-2 molecule screening and the result was presented in Table 5. In the selected peptides pool for HLA binding, Nucleocapsid_104–112_ (LSPRWYFYY) was predicted as both HLA class-I and class II binding peptides. Additionally, this peptide may excel in the capability of binding to a large number of HLA molecules as shown in Tables 4 and 5. A partially overlapping region was found in the CTL epitope Nucleocapsid_66–74_ (FPRGQGVPI) and the helper T-lymphocyte (Th) epitope Nucleocapsid_67–75_ (PRGQGVPIN), which suggests the sequence containing these two epitopes may initiate both CD4^+^ and CD8^+^ dependent immune response.

**Table 4:** HLA class-I alleles binding epitopes predicted by IEDB server

Peptide	Position	HLA class-I alleles	Vaxijen score
LSPRWYFYY	104–112	HLA-A*0101, HLA-A*3002, HLA-B*5701, HLA-A*1101, HLA-A*2601, HLA-B*5801, HLA-A*2402, HLA-A*3101, HLA-B*3501, HLA-B*1501, HLA-A*0301, HLA-A*6801	1.2832
RSRNSSRNS	189–197	HLA-A*3001	1.1144
IGYYRRATR	84–92	HLA-A*3101, HLA-A*3301, HLA-A*6801, HLA-A*0301, HLA-A*3001	0.8880
FTALTQHGK	53–61	HLA-A*6801, HLA-A*1101, HLA-A*0301, HLA-A*0101, HLA-A*3001, HLA-A*3101	0.8510
KSAAEASKK	249–257	HLA-A*0301, HLA-A*1101, HLA-A*3001, HLA-A*6801, HLA-A*3101	0.7679
FPRGQGVPI	66–74	HLA-B*0702, HLA-B*5101, HLA-B*5301, HLA-B*0801, HLA-B*3501	0.7585

**Table 5:** HLA class-II alleles binding epitopes predicted by IEDB server

Peptides	Position	HLA class-II alleles	Vaxijen score
IKLDDKDPN	337–345	DRB1_0701, DRB1_0301, DRB3_0101, DRB1_0405, DPA1_0301/DPB1_0402, DQA1_0101/DQB1_0501, DRB1_0901, DRB1_1101, DRB5_0101, DRB4_0101, DRB1_0101, DRB1_1302, DQA1_0501/DQB1_0301, DPA1_0103/DPB1_0201,	2.3118
RSGARSKQR	32–40	DRB5_0101, DRB4_0101, DQA1_0501/DQB1_0301	1.7874
RIGMEVTPS	319–327	DRB1_1101, DRB1_0401, DRB1_0405, DQA1_0102/DQB1_0602, DRB1_0802, DRB1_0301, DPA1_0301/DPB1_0402, DRB4_0101, DQA1_0501/DQB1_0301, DRB1_0901, DRB5_0101, DQA1_0401/DQB1_0402, DRB1_0101, DRB1_0701, DQA1_0501/DQB1_0201, DRB1_1501, DPA1_0201/DPB1_0101, DPA1_0103/DPB1_0201	1.5314
RGTSPARMA	203–211	DQA1_0501/DQB1_0301, DRB1_0901, DQA1_0102/DQB1_0602	1.2953
LSPRWYFYY	104–112	DRB1_0405, DQA1_0101/DQB1_0501, DPA1_0103/DPB1_0201, DPA1_0201/DPB1_0501, DRB3_0101, DRB1_1201, DRB1_1302, DPA1_0201/DPB1_0101, DRB1_0901, DQA1_0501/DQB1_0201, DRB1_1501, DRB1_1101, DRB1_0101, DRB1_0401, DRB1_0701, DPA1_0301/DPB1_0402, DRB5_0101, DRB1_0301	1.2832
PRGQGVPIN	67–75	DQA1_0501/DQB1_0301, DRB1_1302, DRB1_0101, DRB1_0901, DRB1_0401, DRB1_0701, DRB1_1501, DRB1_0405	1.1707

### Selected T-cell epitopes feature profiling and evaluation

Peptide stability, mutation analysis, toxicity, allergenicity, hydro and physiochemical features were calculated and the results were presented in Supplementary Table 6. While no peptide listed is toxic, a majority of them are potentially allergenic. To forecast the probability of an immune response induced by the predicted HLA-1 binding peptides, the class I immunogenicity test was performed and the scores were presented in Table 6. A higher score indicates a higher potential of immune response induction.

**Table 6:** Class-I immunogenicity

Peptide	Position	Class-I immunogenecity
LSPRWYFYY	104–112	0.35734
RSRNSSRNS	189–197	0.1499
IGYYRRATR	84–92	−0.00164
FTALTQHGK	53–61	−0.0226
KSAAEASKK	249–257	−0.07922
FPRGQGVPI	66–74	−0.26664

### Peptides selected and multi-epitope based vaccine design

**Fig. 5:**
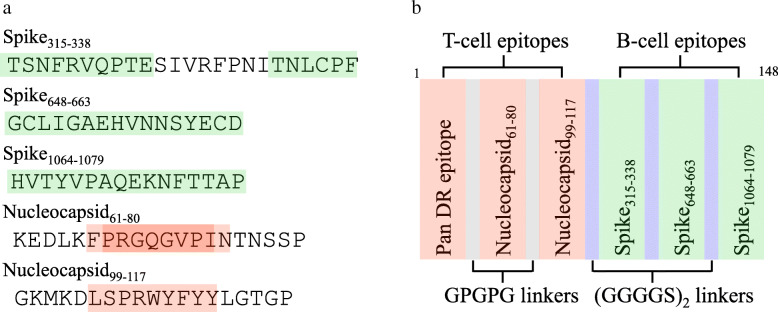
Epitope based vaccine design. **a** B-cell epitopes (highlighted in light green color) and T-cell epitopes (highlighted in pink color) presented in the selected peptides. **b** Schematic view of vaccine construct

To induce humoral and cellular immune response simultaneously, five peptides that contain four linear B-cell epitopes and three T-cell epitopes (Fig. 5a) were selected for vaccine development. To facilitate the processing of the T-cell epitopes, selected peptides from nucleocapsid protein were extended five amino acid residues at both N- and C-terminus as compared to the predicted epitopes. These peptides were scanned on the IEDB database. We found that five sequences presented in the vaccine construct were identical to the experimentally verified epitopes on SARS-CoV. These determined peptides displayed a strong or medium binding affinity to a series of MHC molecules (Supplementary Table 7). These epitopes likely possess cross-protective effects against SARS-CoV-2 as well. As shown in Fig. 5b, peptides selected in this study were joined together by using GPGPG and (GGGGS)_2_ linkers. The Pan DR epitope (PADRE), a universal Th epitope that activates CD4^+^ cells [31], was introduced at the N terminus of the vaccine construct to enhance helper T cell activity. The vaccine construct can be generated as previously reported [32, 33].

## Discussion

Different from the less harmful human coronaviruses continuously circulating in the human population, coronaviruses originated from animals could become fatal pathogens by crossing species barriers. SARS-CoV in 2003, MERS-CoV since 2012, and SARS-CoV-2 right now all have caused large-scale epidemic problems. Effective and economic preventive approaches are in need urgently at the current situation of pandemic.

Compared to traditional vaccine development, potent epitopes can be predicted via bioinformatics analysis, which makes the vaccine design straightforward and fast [34]. As the majority of spike protein is exposed outside the virion, it could be an ideal target to search for B-cell epitopes. Spike proteins in MERS-CoV and SARS-CoV have been shown to induce robust immune response [35, 36]. Baruah and Bose’s study also indicated that epitopes on spike protein could be promising candidates for SARS-CoV-2 vaccine development [37]. In another study, the experimentally determined epitopes derived from SARS-CoV were screened and those harboring the same sequence in SARS-CoV-2 were identified [38]. Similar strategy was followed in Grifoni’s study [39]. Though the SARS-CoV-2 spike protein displayed 77.38% sequence identity toward that of SARS-CoV, most of the antibodies against SARS-CoV spike protein showed poor cross-reactivity with that of SARS-CoV-2 [40], which indicates the spike proteins have significantly variable structure. Thus, we applied distinct strategy and evaluated the surface accessibility of predicted B-cell epitopes to exclude those buried inside the protein. Though we utilized similar bioinformatics tools and resources, the peptides we selected for vaccine development are not alike. In our analysis of SARS-CoV-2 spike protein, four peptides identified from multi-step screening displayed excellent surface accessibility. The peptide Spike_333–338_ produces a random coil structure and has a high antigenicity score. Noticeably, this peptide sits in the receptor-binding domain (RBD) of spike protein of SARS-CoV-2, which has been proved to mediate the binding to ACE2 on the epithelial membrane of human lungs [41]. Likely, antibodies recognizing this epitope could also neutralize the virus and prevent infection. The epitope Spike_648–663_ predicted in this study is 12 amino acids upstream of the furin cleavage site which is critical for SARS-CoV-2 biogenesis [42]. We expect that an antibody recognizing the epitope Spike_648–663_ could have expanded therapeutic function.

Besides B-cell epitope prediction on spike protein, we selected nucleocapsid protein as a target protein for T-cell epitope computation for the following reasons. First, nucleocapsid protein in SARS-CoV and MERS-CoV has been experimentally tested as a robust immunogen to induce cytotoxic T lymphocyte response [26, 27]; Second, nucleocapsid protein is the predominant protein expressed in the virion during the early stage of infection [43]; Third, nucleocapsid protein was detected in the majority of SARS-CoV infected patients as early as day 1 of infection [44, 45], which suggests nucleocapsid protein-based vaccine may evoke T-cell dependent immune response timely. Eighty-one and sixty-four peptides within nucleocapsid protein were predicted to bind HLA-1 and HLA-2 molecules respectively. The peptide Nucleocapsid_104–112_ showed an immunogenicity score of 0.36, suggesting a capability to initiate a strong immune response. Moreover, the peptides Nucleocapsid_104–112_ and Nucleocapsid_66–75_ harbor experimentally determined epitopes that bind to a number of HLA-1 molecules. In this study, Nucleocapsid_104–112_ and Nucleocapsid_66–75_ are forecasted as multi-epitopes that bind to both HLA-1 and HLA-2 molecules, suggesting a potency of both CTL and Th-mediated immune response initiation. Though these peptides were predicted to be non-toxic, we noticed that a large number of these peptides could be allergens. Thus, special attention should be paid to potential allergic reactions during the pre-clinical and clinical trials. As nucleocapsid protein is highly conserved between SARS-CoV-2 and SARS-CoV, available information on nucleocapsid protein-based vaccine against SARS-CoV could be helpful.

The final vaccine construct comprising of CTL, Th, and B-cell epitopes is predicted to initiate protective humoral and cellular immune response against SARS-CoV-2.

## Conclusions

B-cell epitopes on spike protein and T-cell epitopes in the nucleocapsid protein were predicted and analyzed in the current study. A total of 63 linear B-cell epitopes on spike protein were forecasted by ABCpred and BepiPred servers. Ten residues within spike protein (Gly^496^, Glu^498^, Pro^499^, Thr^500^, Leu^1141^, Gln^1142^, Pro^1143^, Glu^1144^, Leu^1145^, Asp^1146^) were forecasted by DiscoTope 2.0 program as components of conformational B-cell epitopes. IEDB server was used for T-cell epitopes prediction, which gave rise to 81 and 64 peptides with binding capability to class-I and class-II molecule respectively. Four B-cell epitopes: Spike_315–324_ (TSNFRVQPTE), Spike_648–663_ (GCLIGAEHVNNSYECD), Spike_1064–1079_ (HVTYVPAQEKNFTTAP), and Spike_333–338_ (TNLCPF) were selected from the list based on their antigenicity score and surface accessibility. The T-cell epitopes Nucleocapsid_104–112_ (LSPRWYFYY) and Nucleocapsid_66–75_ (FPRGQGVPIN) on nucleocapsid protein could bind a wide spectrum of both HLA-1 and HLA-2 molecules. The final vaccine construct consists of CTL, Th, and B-cell epitopes that potentially protect individuals against SARS-CoV-2 by inducing both humoral and cellular immune response, which should be successively validated within both in vitro and in vivo models.

## Supplementary information

**Additional file 1.** Supplementary file S1.

**Additional file 2.** Supplementary file S2.

**Additional file 3.** Supplementary figures and tables.

**Additional file 4.** B-cell-epitope-animation.

## Data Availability

All analyzed data of this study are included with the manuscript and its supplementary information files.

## Abbreviations

SARS-CoV: Severe acute respiratory syndrome associated coronavirus
MERS-CoV: Middle East respiratory syndrome coronavirus
HCoV-NL63: Human Coronavirus NL63
HLA: Human leukocyte antigen
MHC: Major histocompatibility complex
WHO: World Health Organization
NSP: Non-structure protein
ORF: Open reading frame
CTL: Cytotoxic T-lymphocyte
Th: Helper T-lymphocyte
