# Supplementary material for: Bioinformatics analysis of epitope-based vaccine design against the novel SARS-CoV-2
Source: Infect Dis Poverty. 2020 Jul 10;9:88. doi: 10.1186/s40249-020-00713-3 (PMC7395940; doi:10.1186/s40249-020-00713-3)
Supplement: Supplementary file 3 — Additional file 3. Supplementary figures and tables. [file 40249_2020_713_MOESM3_ESM.pptx]

## Slide 1
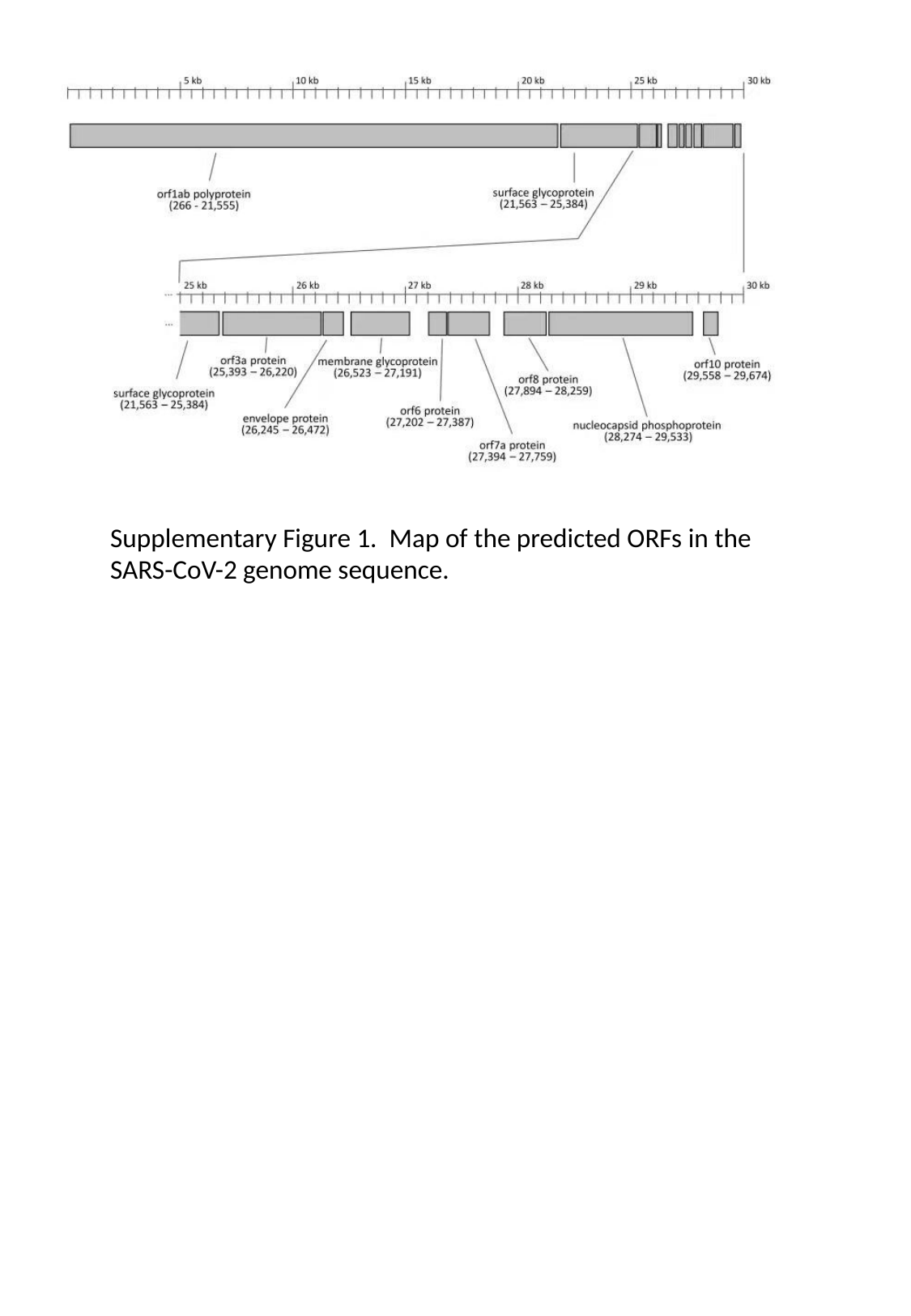

Supplementary Figure 1. Map of the predicted ORFs in the SARS-CoV-2 genome sequence.

## Slide 2
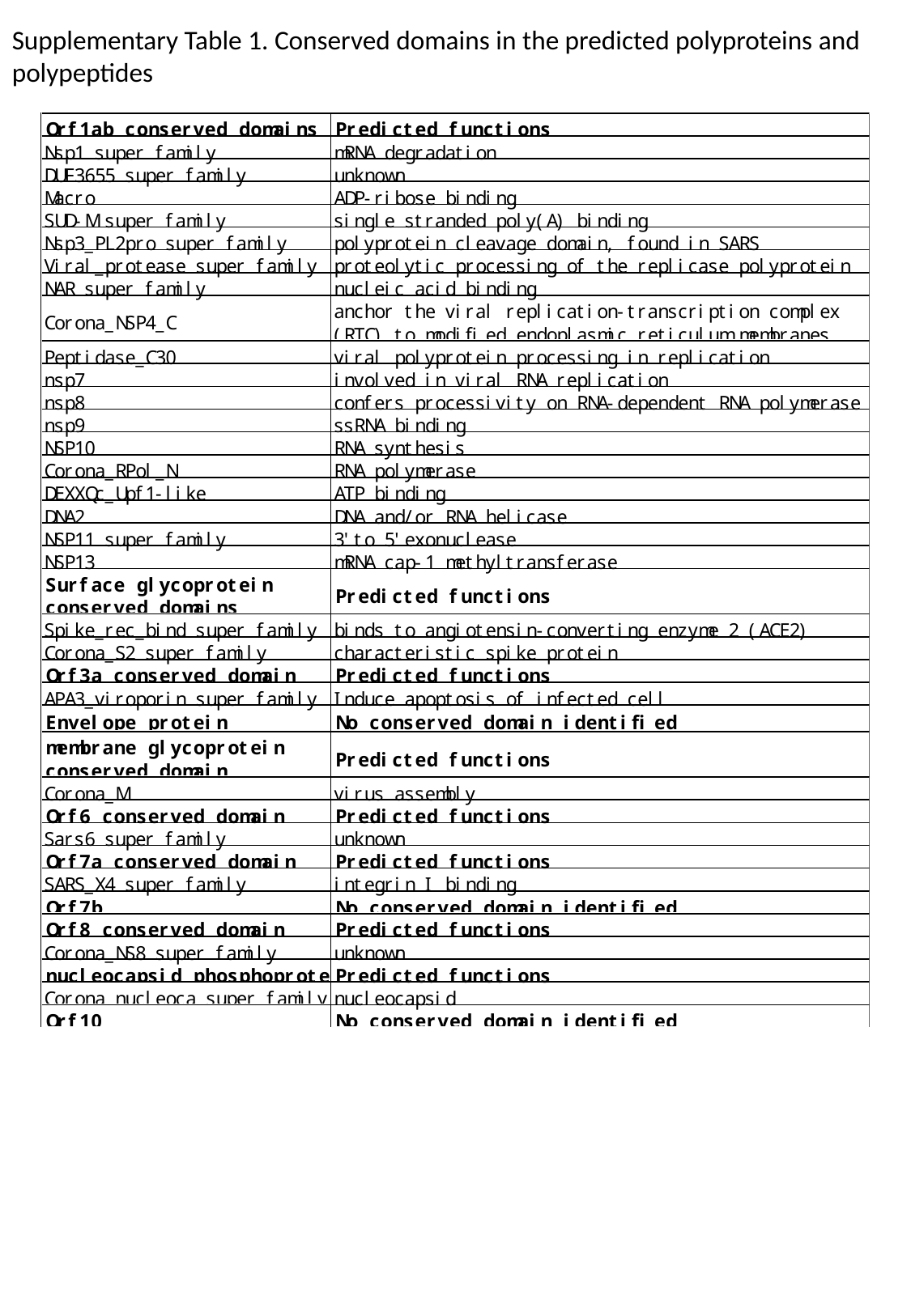

Supplementary Table 1. Conserved domains in the predicted polyproteins and polypeptides

## Slide 3
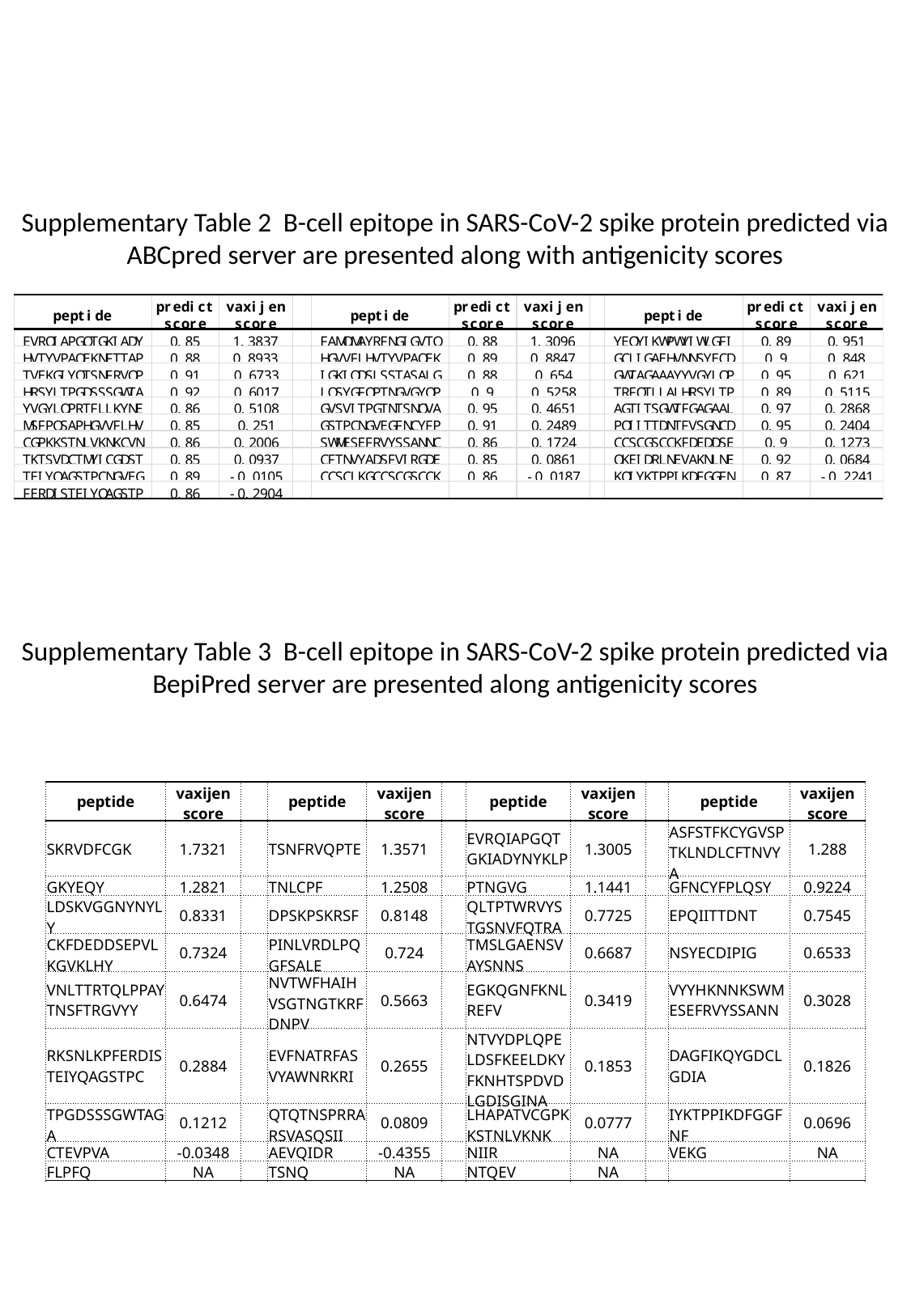

Supplementary Table 2 B-cell epitope in SARS-CoV-2 spike protein predicted via ABCpred server are presented along with antigenicity scores
Supplementary Table 3 B-cell epitope in SARS-CoV-2 spike protein predicted via BepiPred server are presented along antigenicity scores
| peptide | vaxijen score | | peptide | vaxijen score | | peptide | vaxijen score | | peptide | vaxijen score |
| --- | --- | --- | --- | --- | --- | --- | --- | --- | --- | --- |
| SKRVDFCGK | 1.7321 | | TSNFRVQPTE | 1.3571 | | EVRQIAPGQTGKIADYNYKLP | 1.3005 | | ASFSTFKCYGVSPTKLNDLCFTNVYA | 1.288 |
| GKYEQY | 1.2821 | | TNLCPF | 1.2508 | | PTNGVG | 1.1441 | | GFNCYFPLQSY | 0.9224 |
| LDSKVGGNYNYLY | 0.8331 | | DPSKPSKRSF | 0.8148 | | QLTPTWRVYSTGSNVFQTRA | 0.7725 | | EPQIITTDNT | 0.7545 |
| CKFDEDDSEPVLKGVKLHY | 0.7324 | | PINLVRDLPQGFSALE | 0.724 | | TMSLGAENSVAYSNNS | 0.6687 | | NSYECDIPIG | 0.6533 |
| VNLTTRTQLPPAYTNSFTRGVYY | 0.6474 | | NVTWFHAIHVSGTNGTKRFDNPV | 0.5663 | | EGKQGNFKNLREFV | 0.3419 | | VYYHKNNKSWMESEFRVYSSANN | 0.3028 |
| RKSNLKPFERDISTEIYQAGSTPC | 0.2884 | | EVFNATRFASVYAWNRKRI | 0.2655 | | NTVYDPLQPELDSFKEELDKYFKNHTSPDVDLGDISGINA | 0.1853 | | DAGFIKQYGDCLGDIA | 0.1826 |
| TPGDSSSGWTAGA | 0.1212 | | QTQTNSPRRARSVASQSII | 0.0809 | | LHAPATVCGPKKSTNLVKNK | 0.0777 | | IYKTPPIKDFGGFNF | 0.0696 |
| CTEVPVA | -0.0348 | | AEVQIDR | -0.4355 | | NIIR | NA | | VEKG | NA |
| FLPFQ | NA | | TSNQ | NA | | NTQEV | NA | | | |

## Slide 4
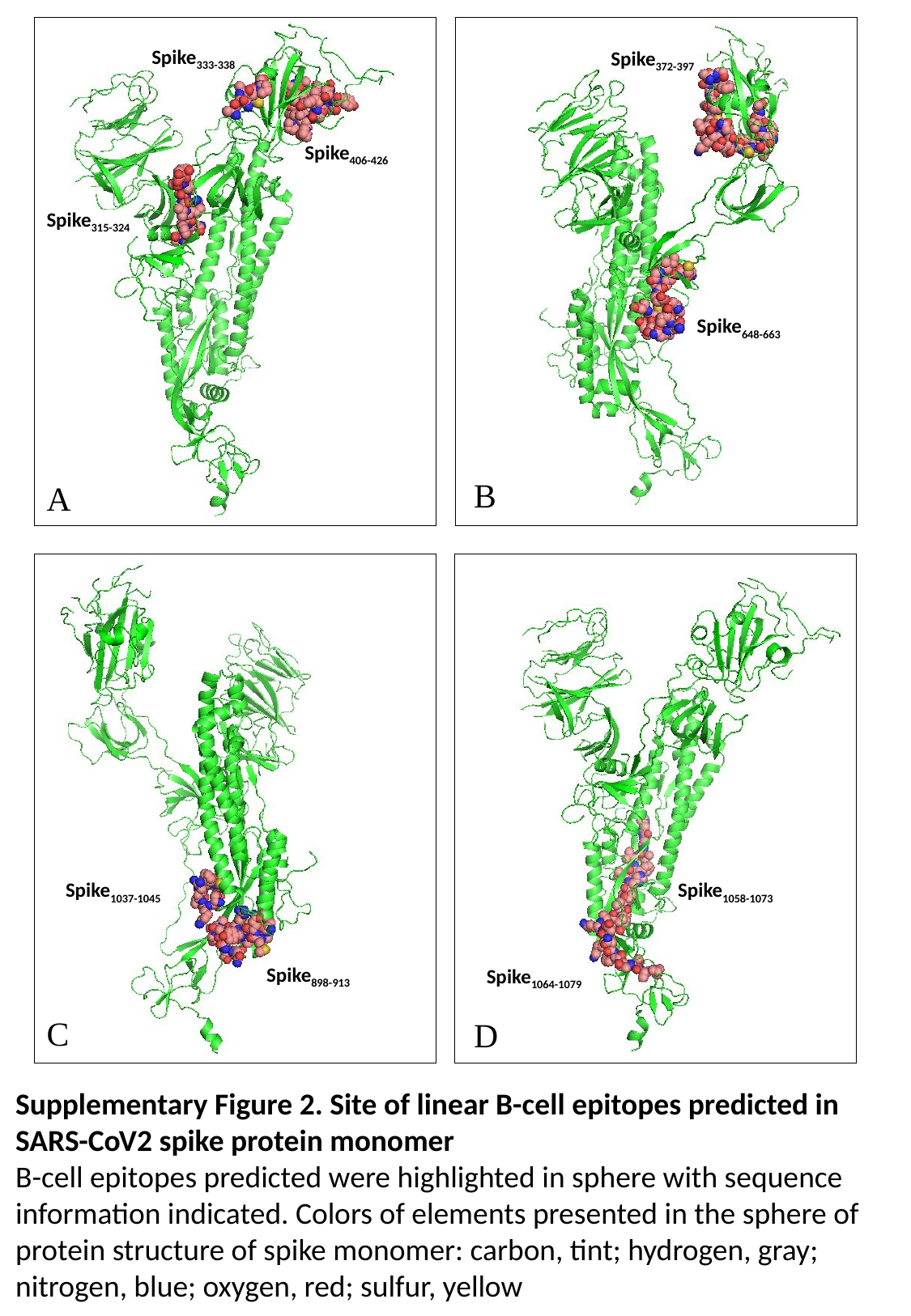

Spike333-338
Spike372-397
Spike406-426
Spike315-324
Spike648-663
B
A
Spike1037-1045
Spike1058-1073
Spike898-913
Spike1064-1079
C
D
Supplementary Figure 2. Site of linear B-cell epitopes predicted in SARS-CoV2 spike protein monomer
B-cell epitopes predicted were highlighted in sphere with sequence information indicated. Colors of elements presented in the sphere of protein structure of spike monomer: carbon, tint; hydrogen, gray; nitrogen, blue; oxygen, red; sulfur, yellow

## Slide 5
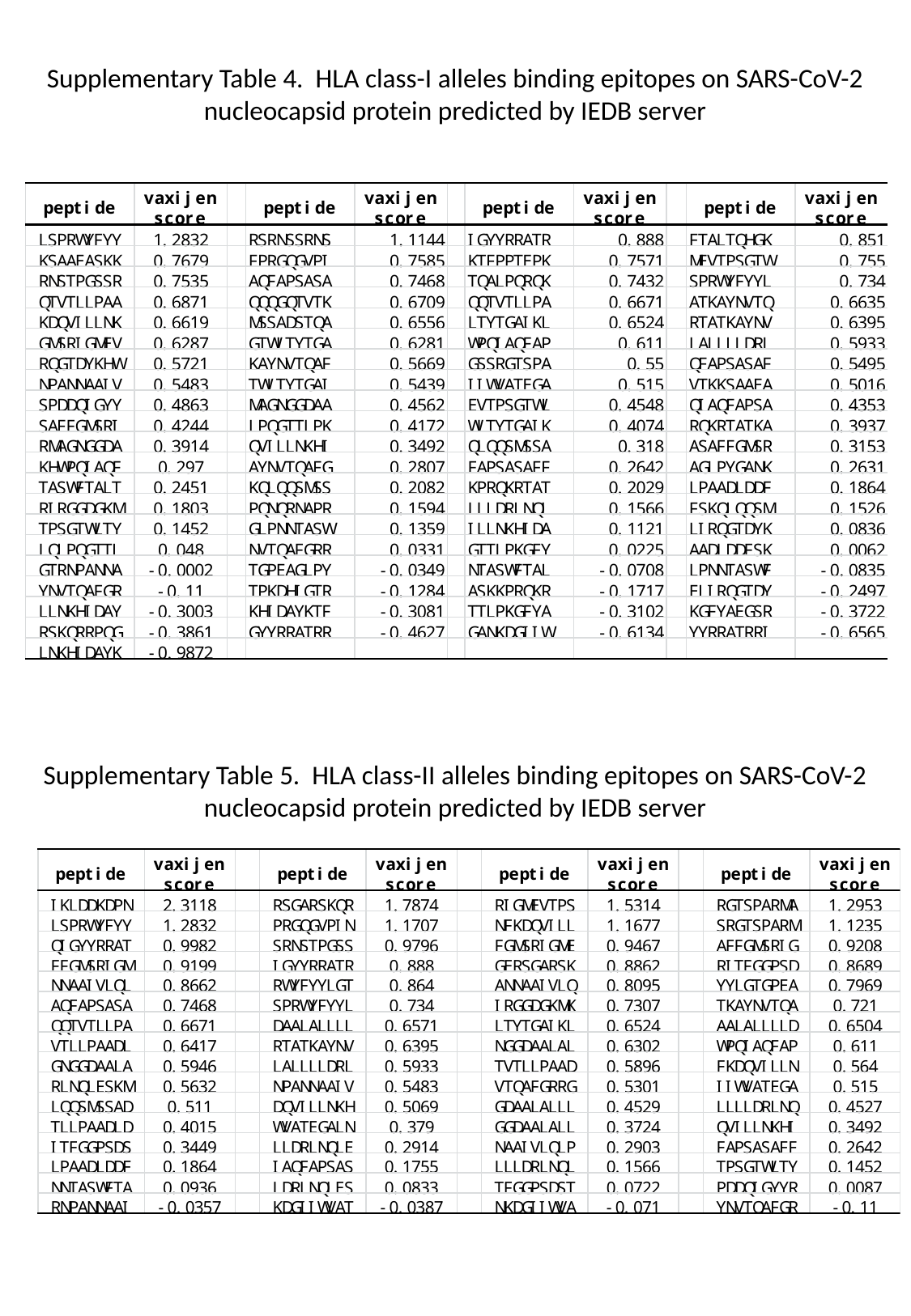

Supplementary Table 4. HLA class-I alleles binding epitopes on SARS-CoV-2 nucleocapsid protein predicted by IEDB server
Supplementary Table 5. HLA class-II alleles binding epitopes on SARS-CoV-2 nucleocapsid protein predicted by IEDB server

## Slide 6
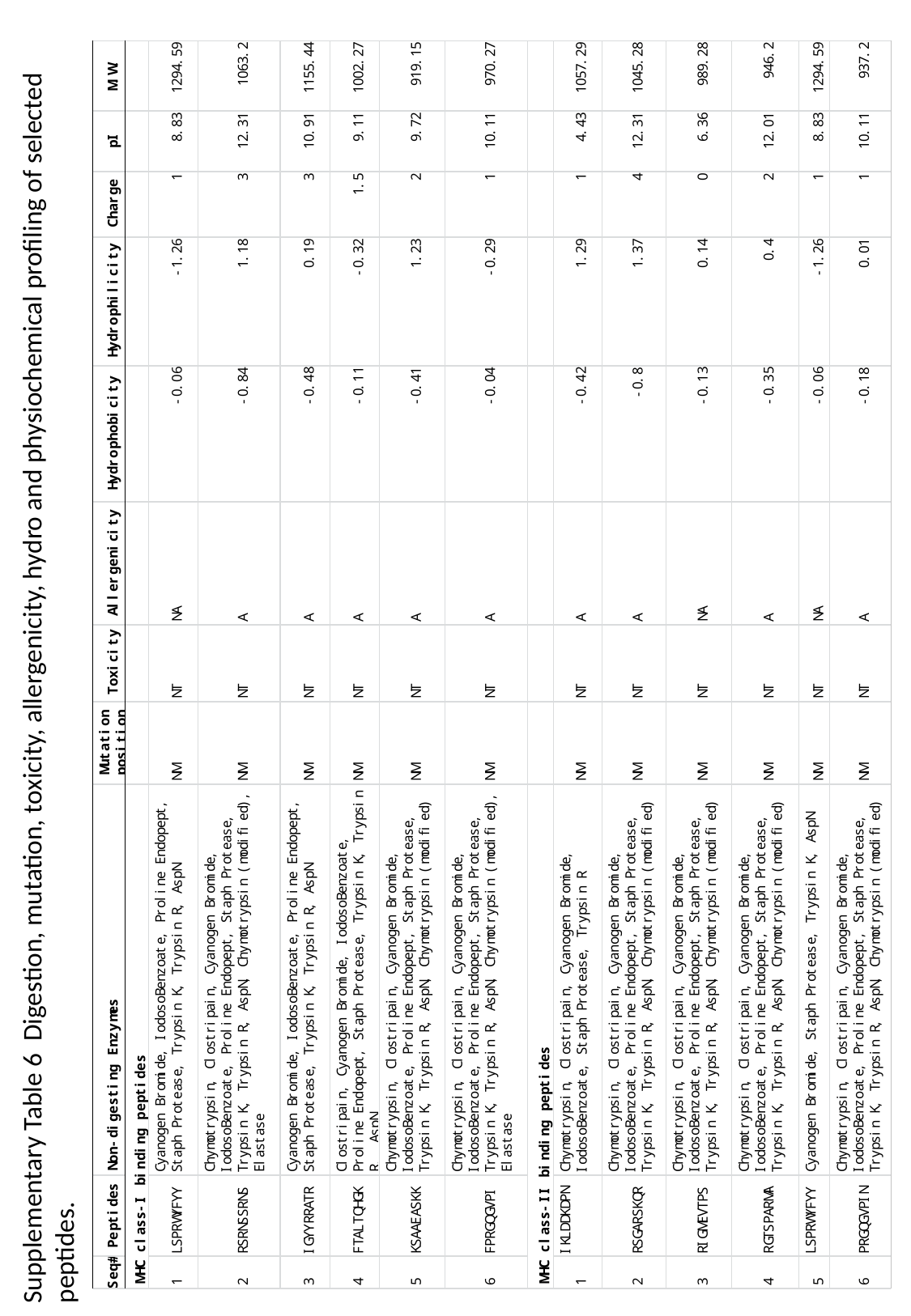

Supplementary Table 6 Digestion, mutation, toxicity, allergenicity, hydro and physiochemical profiling of selected peptides.

## Slide 7
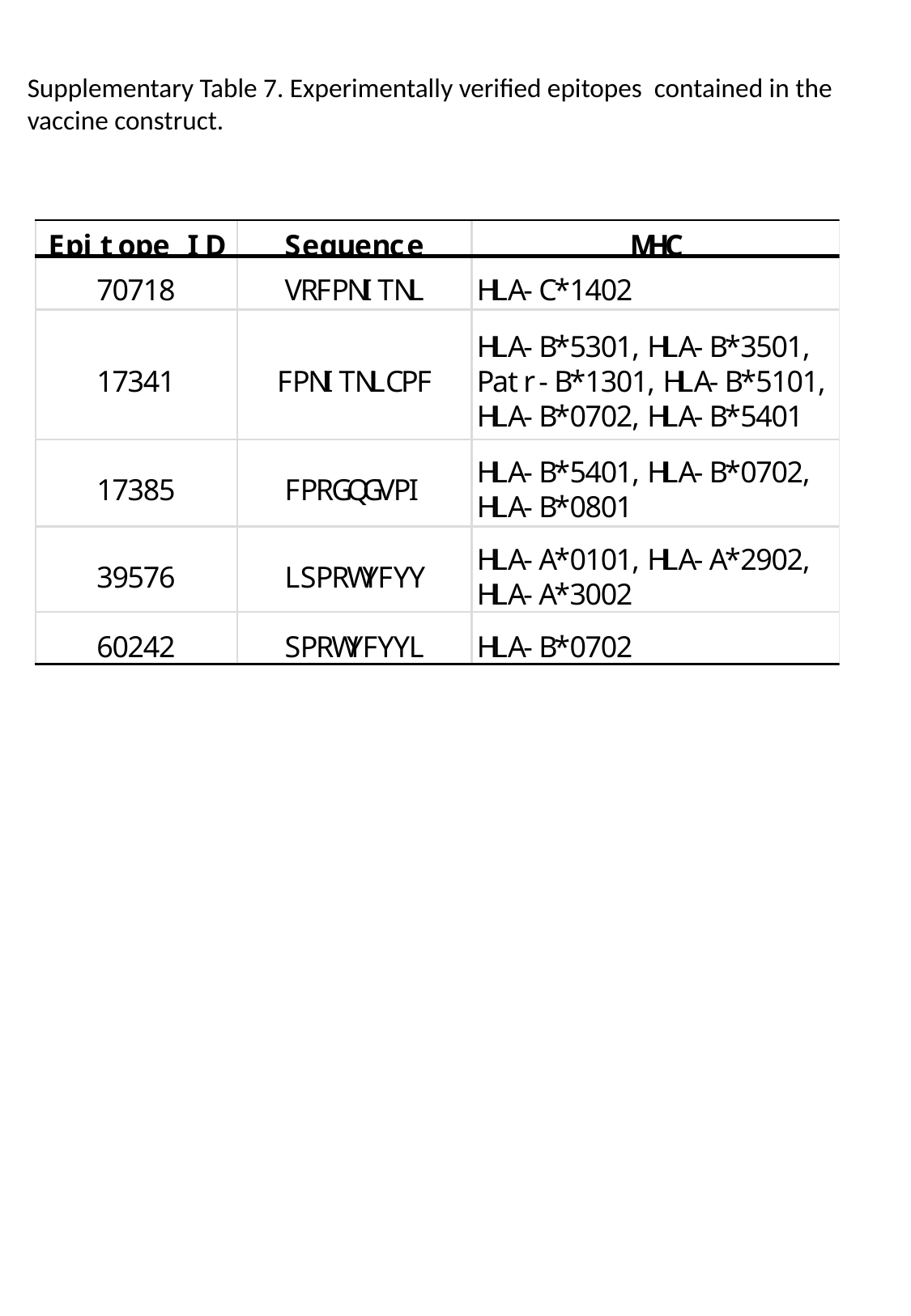

Supplementary Table 7. Experimentally verified epitopes contained in the vaccine construct.
