# Supplementary material for: Bioinformatics analysis of epitope-based vaccine design against the novel SARS-CoV-2
Source: Infect Dis Poverty. 2020 Jul 10;9:88. doi: 10.1186/s40249-020-00713-3 (PMC7395940; doi:10.1186/s40249-020-00713-3)
Supplement: Supplementary file 4 — Additional file 4. B-cell-epitope-animation. [file 40249_2020_713_MOESM4_ESM.pptx]

## Slide 1
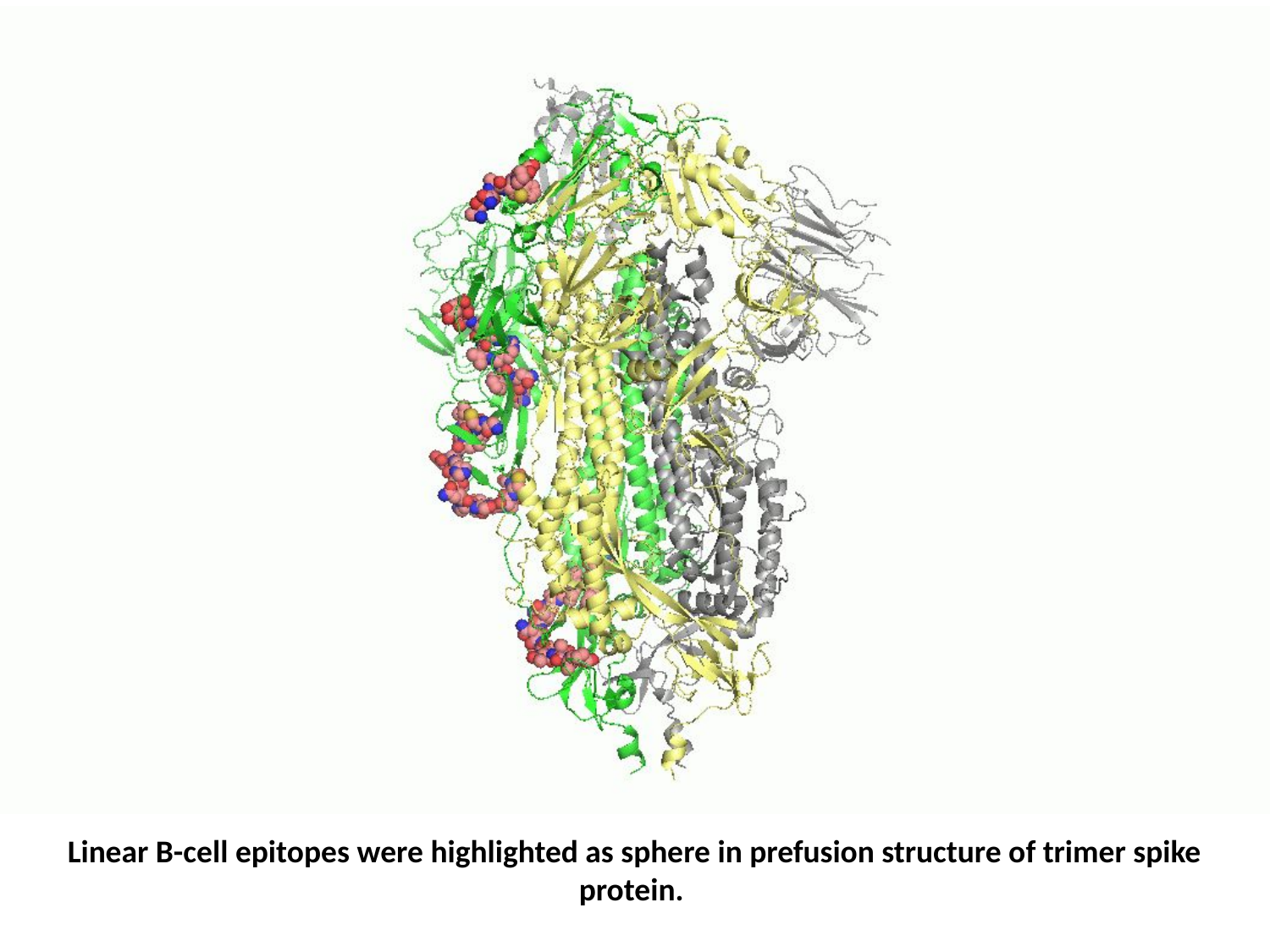

Linear B-cell epitopes were highlighted as sphere in prefusion structure of trimer spike protein.
